# Maspin enhances cisplatin chemosensitivity in bladder cancer T24 and 5637 cells and correlates with prognosis of muscle-invasive bladder cancer patients receiving cisplatin based neoadjuvant chemotherapy

**DOI:** 10.1186/s13046-015-0282-y

**Published:** 2016-01-06

**Authors:** Jinbo Chen, Long Wang, Yunhua Tang, Guanghui Gong, Longfei Liu, Minfeng Chen, Zhi Chen, Yu Cui, Chao Li, Xu Cheng, Lin Qi, Xiongbing Zu

**Affiliations:** Department of Urology, Xiangya Hospital, Central South University, Changsha, Hunan 410008 China; Department of Pathology, Xiangya Hospital, Central South University, Changsha, Hunan 410008 China

**Keywords:** Maspin, Muscle invasive bladder cancer, Cisplatin, Chemosensitivity

## Abstract

**Background:**

Maspin, a non-inhibitory member of the serine protease inhibitor superfamily, has been characterized as a tumor suppressor gene in multiple cancer types. Chemotherapeutic insensitivity is one of major obstacles to effectively treating muscle invasive bladder cancer (MIBC). This study was conducted to investigate the role and probable mechanism of Maspin enhancing cisplatin chemosensitivity of bladder cancer in vitro and MIBC patients.

**Methods:**

Maspin expression was quantified by qRT-PCR in two MIBC cell lines (T24 and 5637). After successful established Maspin overexpression model by lipidosome transfection, MTT and cell apoptosis assay were used to assess the MIBC’s cisplatin sensitivity. Western blot method was used to test PI3K/ AKT/mTOR signal passway and apoptosis related molecules Caspase3 and Bcl-2. Additionally, we evaluated Maspin expression and prognosis in 62 MIBC cases who underwent cisplatin based neoadjuvant chemotherapy (NACT) using immunohistochemistry.

**Result:**

Upregulate Maspin expression could enhance the chemosensitivity induced by cisplatin in T24 and 5637 cell lines. The cell viability, cloning ability and IC50 were reduced while apoptosis rate was upregulated when cells were transfected Maspin. Phospho(p)-AKT, PI3K, mTOR, and Bcl-2 expression were significantly decreased, whereas Caspase3 was greatly increased in the Maspin group. In the clinic study, there was significant correlation between Maspin expression and overall survival (OS) and progression-free survival (PFS) rate in MIBC patients who received cisplatin based NACT.

**Conclusion:**

Maspin could enhance cisplatin chemosensitivity in T24 and 5637 cell lines. Its expression correlated with prognosis of MIBC patients who received cisplatin based neoadjuvant chemotherapy.

## Background

Bladder cancer is one of the most common types of urinary tract malignancy, with an estimated 429,800 new cases of bladder cancer and 165,100 deaths occurred in 2012 worldwide. Incidence rates are highest in Europe, Northern America, Western Asia, and Northern Africa [1]. It is clinically characterized by its progression, recurrence, metastasis, and drug resistance [2]. At initial diagnosis, approximately 70 % of cases are diagnosed as non-muscle invasive bladder cancer (NMIBC) and approximately 30 % as muscle-invasive bladder cancer (MIBC) [3]. Although the majority of patients presenting with superficial disease have a favorable prognosis, approximately 70 % of these develop disease recurrence and 10 to 20 % of these patients progress to MIBC [4, 5]. The standard treatment for MIBC is radical cystectomy, which only provides a mere five-year survival rate of 50 % [6]. In order to improve the effects of treatment, perioperative chemotherapy has been explored since the 1980s. Although cisplatin is the first-line chemotherapy for advanced bladder cancer, the cisplatin/gemcitabine (GC) regimen has a median time-to-progression of only six months and has no effect on overall survival after radical cystectomy in high-risk patients [7]. Cancer cell resistance to cisplatin is a major obstacle to the effective treatment of bladder cancer, and the underlying mechanisms of the resistance are unclear till now.

Maspin (mammary serine protease inhibitor), is a member of the serine protease inhibitor/non-inhibitor superfamily. Recent studies showed it was a tumor suppressor gene in multiple cancers. Its effects included inhibition of cancer cell invasion, attachment to extracellular matrices, increased sensitivity to apoptosis, and inhibition of angiogenesis [8, 9]. Maspin expression was found in high quantities in normal urothelium. It was preserved in superficial bladder cancer but significantly diminished in invasive carcinoma. And within the group of invasive cancers, it was found that maspin expression was associated with good prognosis [10]. There were some reports of Maspin enhancing chemosensitivity in ovarian and breast carcinoma cells [11, 12]. Also, clinic relevance of Maspin expression and adjuvant chemotherapy prognosis in advanced gastric cancer was confirmed [13]. But its role and mechanism in chemosensitivity of bladder cancer remain unclear. So, in the current study, we investigated the role and probable mechanism of Maspin enhancing the cisplatin chemosensitivity of bladder cancer in vitro and MIBC patients.

## Methods

### Cell culture

The MIBC cell lines T24, 5637 and human bladder epithelial cell line SV-HUC-1 were obtained from American Type Culture Collection (ATCC). Cells were cultured in 1640 medium (Invitrogen, Carlsbad, CA) with 10 % FBS (Invitrogen) and 1 % penicillin/streptomycin (Invitrogen). All cells were cultured in a sterile incubator maintained with 5 % CO2 at 37 °C.

### Real-time RT-PCR

Total RNA was extracted from cells with Trizol reagent (Invitrogen) according to the manufacturer’s instructions. The relative expression level of Maspin was determined by quantitative real-time RT-PCR using standard SYBR Green RT-PCR Kit (Takara, Otsu, Japan). The specific primer pairs are as follows: Maspin sense, 5′-CTGACAACAGTGTGAACGAC-3′, antisense, 5′-CAAGCCTTGGGATCAATC ATCT-3′; β-actin as an internal control, sense, 5′-AGGGGCCGGACTCGTCATAC T-3′ and antisense, 5′-GGCGGCACCAC CATGTACCCT-3′. The relative expression of Maspin mRNA was quantified using the GraphPad Prism 4.0 software (GraphPad Software, San Diego, CA, USA) and 2^-ΔΔCt^ method.

### Western blot analysis

Protein levels were quantified by Bradford assay. The protein sample was diluted, heated for denaturation, and then subjected to dodecyl sulfate polyacrylamide gel electrophoresis (SDS-PAGE) and transferred onto polyvinylidene fluoride membranes (PVDF, Millipore). The membrane was blocked in 0.1 % Triton X-100 and 5 % non-fat milk powder in phosphate-buffered saline for 1 h at 4 °C and then probed with β-actin (1:2000, Santa Cruz Biotechnology, Santa Cruz, CA) rabbit polyclonal primary antibody. After washing 3 times with Tris-buffered saline Tween (TBST), the membrane was incubated in peroxidase-conjugated goat anti-rabbit IgG antibody (1:4000, Immunoway, USA). Bands were visualized by an enhanced chemiluminescence detection system using medical X-ray films and quantified by IPP6.0 analysis software. The intensities of band of interest were expressed relative to the β-actin intensities from the same sample.

### Transfection

Cells were trypsin-digested and seeded onto six-well plates the day prior to the transfection to ensure 60–70 % cell confluence on the day of transfection. pcDNA3.1-Maspin recombinant plasmids were constructed (Data not shown) and transfected into T24 and 5637 cell lines using Lipofectamine 2000 (Invitrogen) according to the manufacturer’s recommendations. pcDNA3.1 without Maspin plasmids were also transfected into these two cell lines as negtive control (NC) groups.

### Cell proliferation assay

Cells in exponential growth were plated at a concentration of 3 × 10^3^ cells/well in 96-well plates for 24 h before use. The culture medium was replaced with fresh medium containing cisplatin with different concentrations for 72 h. Next, MTT (20 μl/well) was added to each well. After 2 h further incubation, the optical density at 570 nm (OD570) of each well was measured with an ELISA reader (ELX-800, Bio-Tek). The inhibition rate for every concentration and the IC50 (the concentration of drug that results in 50 % of the control value) were calculated using Graphpad5 software.

### Cell apoptosis assay

Forty-eight hours after transfection, all group cells were seeded at a density of 5x10^5^ cells per 10-mm dish, and treated with cisplatin at proper concentration for 24 h, respectively. Then the cells were collected by brief trypsinization and washed twice in PBS. After incubation with 5 μl of Annexin V-FITC and 10 μl of propidium iodide (PI) at room temperature for 15 min in dark, cells were analyzed by flow cytometry (BD, NJ, USA). Apoptosis cells were recognized as high Annexin V fluorescence signal with low PI signal. The percentages of apoptotic cells were calculated by data from FACS analysis.

### Colony-formation assay

For all groups, 3 ml complete medium containing 150 cells were added to each well of a six-well plate. Plates were incubated at 37 °C, 5 % CO_2_ for 14 days. After that, cells were gently washed and stained with Giemsa. Colonies containing at least 50 cells were counted.

### Patients and tumor samples

From January 2010 to January 2014, 62 MIBC patients who received cisplatin based neoadjuvant chemotherapy (NACT) and laparoscopic radical cystectomy and pelvic lymph node dissection (RC-PLND) in our center were included in this study. We obtained ethics approval from the ethics committee at Xiangya Hospital, Central South University. Written informed consent was obtained from all participants in our study. Table 1 showed details of patients and tumor characteristics. Follow-up information was available for all 62 patients with a follow-up time of 36.1 (range from 16 to 64) months. Tumor staging was determined by 2009 UICC-TNM staging system and tumor grading was according to 1973 WHO grading system. The chemotherapy of GC regimen were as follows: gemcitabine 1000 mg/m^2^, 1d-1, 8d −1; cisplatin 70 mg/m^2^, 2d −1. a 21-day cycle for 3–4 cycles. NACT was administered GC regimen within 16 weeks before surgery.

**Table 1:** Patient Characteristics

Total patients (n)	62
Age (year)	60.5 (39–78)
Gender (n, %)	
Male	43 (69.4 %)
Female	19 (30.6 %)
Grade (n, %)	
Grade 1	4 (6.5 %)
Grade 2	30 (48.4 %)
Grade 3	28 (45.1 %)
TNM stage (n, %)	
pT2N0	14 (22.6 %)
pT2N+	5 (8.1 %)
pT3N0	11 (17.7 %)
pT3N+	16 (25.8 %)
pT4aN0	3 (4.8 %)
pT4aN+	13 (21.0 %)
Follow-up time (months)	36.1 (16–64)

### Tissue immunohistochemistry

Immunohistochemical (IHC) staining for Mapsin protein was carried out using an SP ready-to-use kit (Zhongshan Jinqiao Biotechnology, Zhongshan, China) according to the manufacturer’s recommendation. Briefly, deparaffinization and rehydration were done before paraffin-embedded sections (4 *μ*m thick) were immersed in 0.01 Mcitric buffer (pH 6.0). The slides were then incubated with 3 % hydrogen peroxide for 10 min, followed by incubation in normal goat serum for 10 min at room temperature. Maspin antibody (monoclonal mouse antibody; NCL-Maspin, Novocastra Laboratories Ltd.) was used at a dilution of 1: 200 as the first antibody according to the manufacturer’s instructions. Sections were then incubated with goat anti-polyvalent antibody for 10 min and subsequently with streptavidin peroxidase for 10 min. The slides were visualized using diaminobenzidine and counterstained with hematoxylin before microscopy.

The IHC score was assessed independently by two surgical pathologists. The number of immunoreactive cells was quantified as ‘proportion score’, including 0; no immunoreactive cells, 1; the numbers of immunoreactive cells were less than 1 %, 2; less than 10 %, 3; less than one-thirds, 4; less than two-thirds, 5; more than two-thirds. The highest number of immunoreactive tumor cells was graded as ‘intensity score’, including 0; no immunoreactivity, 1; weak staining intensity, 2; intermediate, 3; strong. The IHC results were grouped based on the IHC score (proportion score plus intensity score): 0–2, negative(−); 3–8, positive(+). In case of disagreement, the score was reevaluated and consensus was reached [14].

### Statistical analysis

Results were expressed as the means ± standard deviation (SD) from at least three separate experiments. Statistical analysis was carried out using SPSS 16.0 software. Student’s t-test was used to compare the relative amounts of mRNAs and proteins. Survival curves were plotted by the Kaplan–Meier method, and differences were examined by the log-rank test. A value of *P* < 0.05 was considered to indicate statistical significance.

## Results

### Different expression of Maspin in bladder cancer and normal bladder epithelial cell lines

**Fig. 1:**
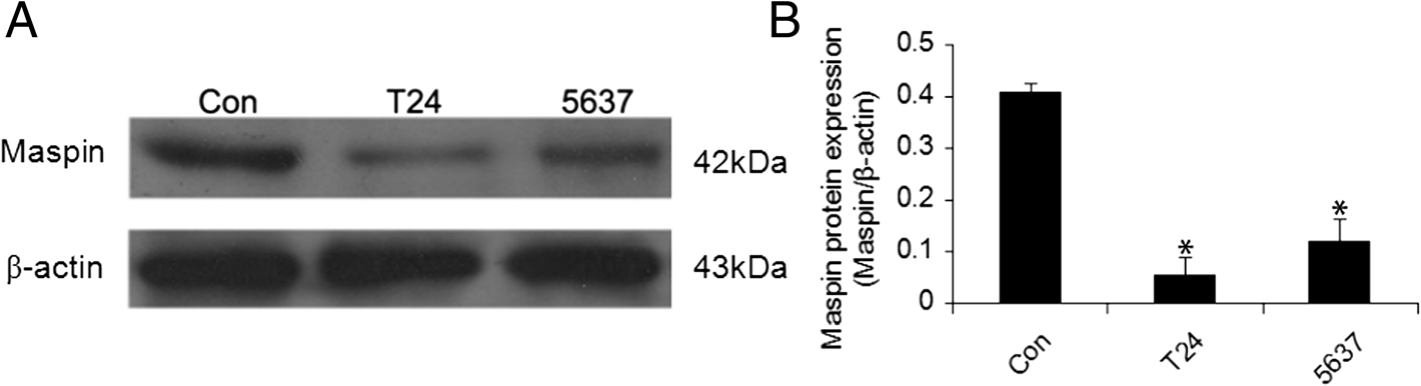
The protein level of Maspin in bladder cancer T24, 5637 and normal bladder epithelial SV-HUC-1 cell lines. Data were shown in the grayscale (**a**) and bar chart (**b**), respectively. Maspin expression was significantly decreased in bladder cancer T24 and 5637 cell lines as compared the control group SV-HUC-1. *P < 0.05. Three independent experiments were performed (*n* = 3)

To delineate the potential role of Maspin in bladder cancer, we compared the protein level in bladder normal and cancer cells. The protein levels of Maspin in MIBC cell lines were determined using western blot. Down-regulation of Maspin was observed in bladder cancer cell lines. The densitometry evaluation of the western blots showed that Maspin expression was significantly decreased in bladder cancer T24 and 5637 cell lines as compared the control group SV-HUC-1, respectively. (*P* < 0.05) (Fig. 1a, b). These results suggest that Maspin may play an important role in malignant progression of bladder cancer.

### Expression levels of Maspin post-transfection in bladder cancer T24 and 5637 cells

**Fig. 2:**
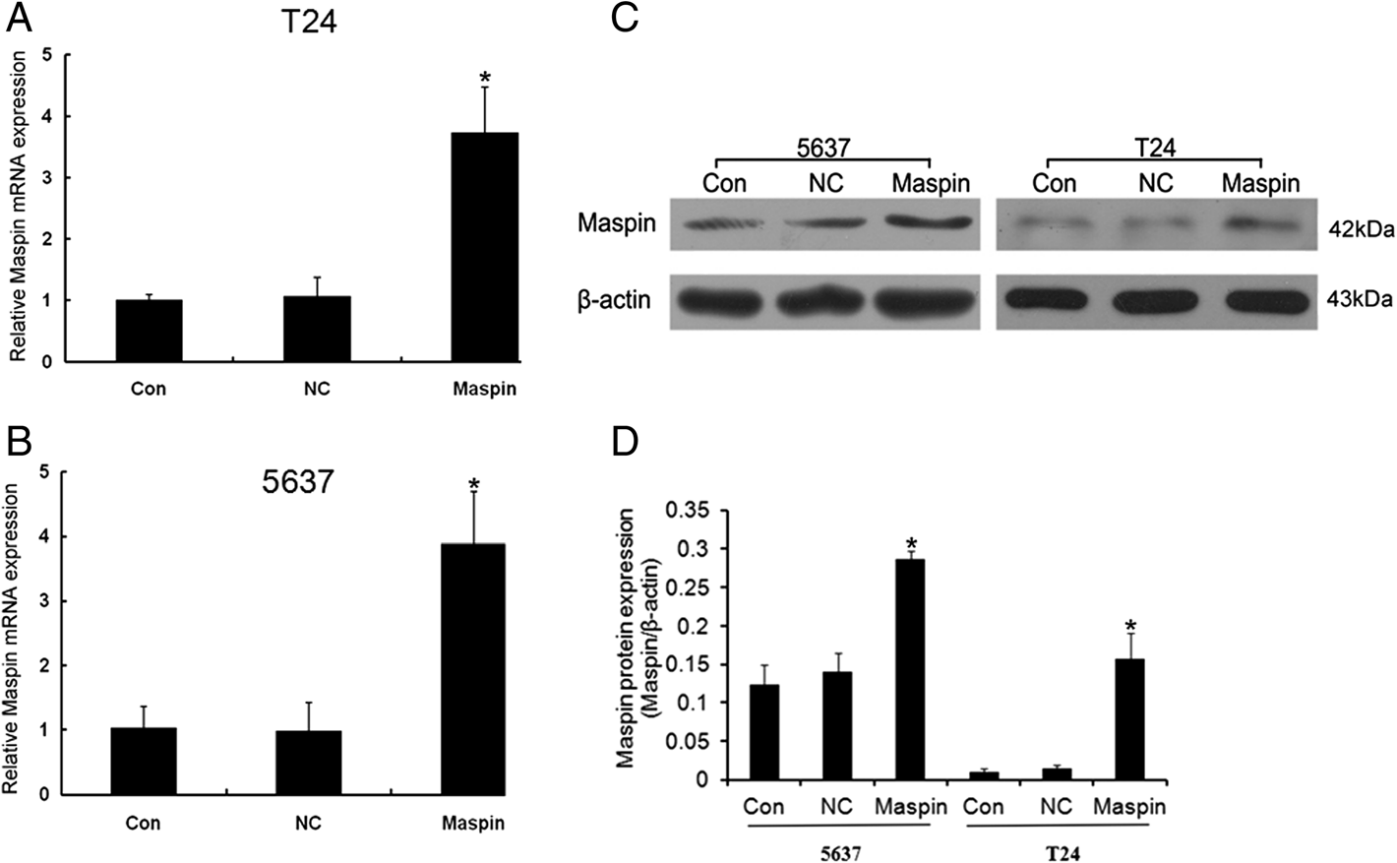
Expression levels of Maspin post-transfection in bladder cancer T24 and 5637 cells. The mRNA and protein levels of Maspin in bladder cancer T24 and 5637 cells transfected with pcDNA3.1-Maspin or negative control (pcDNA3.1-con) were detected by RT-PCR and western blot. **a** and **b** were showed mRNA level of T24 and 5637 cells, respectively. And **c** and **d** were showed protein expression level. *P < 0.05. Three independent experiments were performed (*n* = 3)

To clarify the functions of Maspin in bladder cancer T24 and 5637 cells, gain-of-function cell models were constructed by transfecting with pcDNA3.1-Maspin. Compared with blank control group (Con) and negative control group (NC), Maspin mRNA (Fig. 2a, b) and protein (Fig. 2c, d) level was significantly up-regulated when transfected with pcDNA3.1-Maspin in T24 and 5637 cells (*P* < 0.05).

### Maspin inhibits proliferation and enhancing the sensitivity to cisplatin of bladder cancer T24 and 5637 cells

**Fig. 3:**
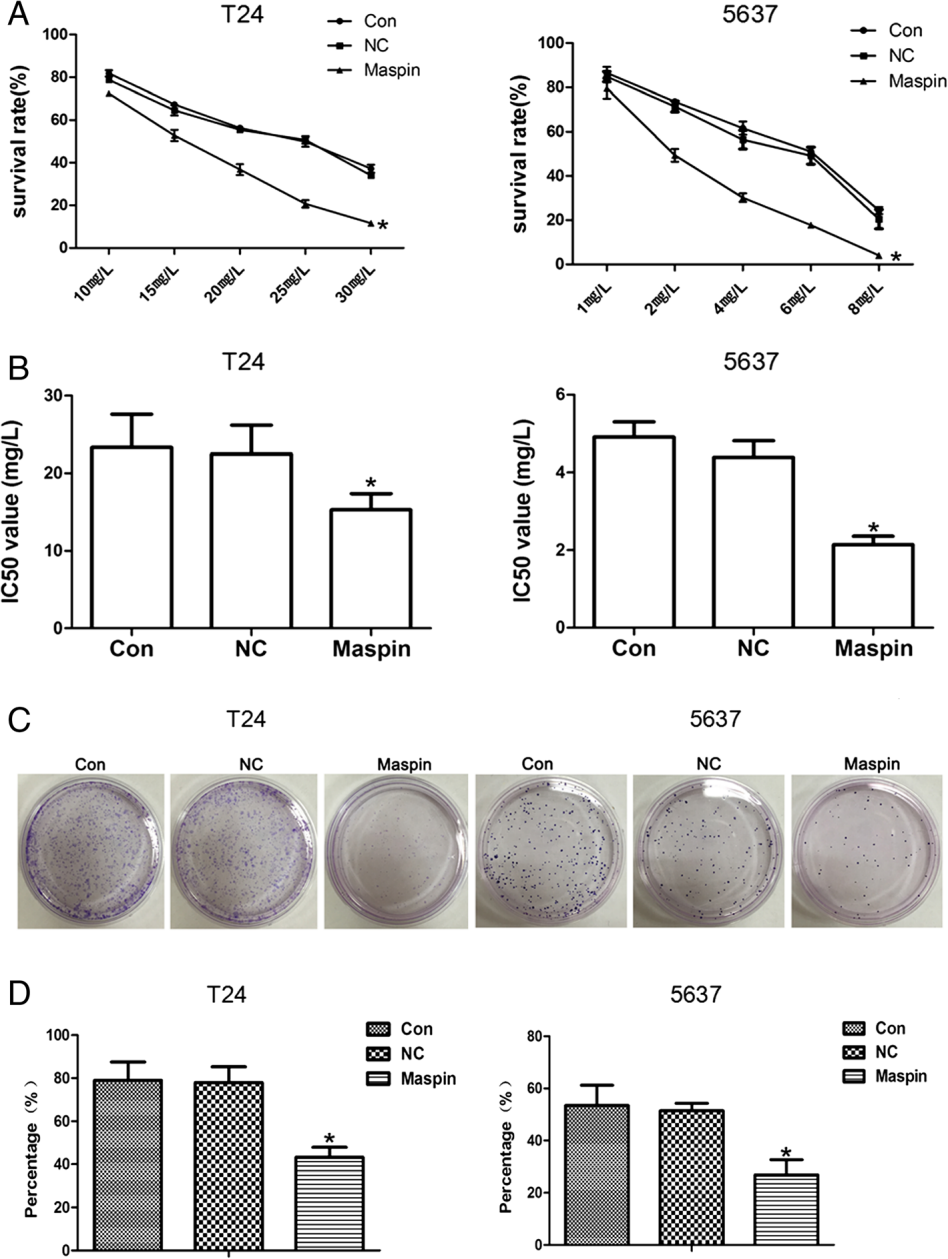
Effects of Maspin on proliferation and sensitivity to cisplatin of T24 and 5637 cells. **a** was showed the cell viability with different cisplatin measured by MTT assay. **b** was showed the IC50 value in each group. Cloning efficiency was calculated after cultured for 2 weeks. **c** and **d** showed the representative results of colony formation assay. Overexpression of Maspin dramatically decreased the cell viability, IC50 value and clone-formation, respectively. *P < 0.05. Three independent experiments were performed (*n* = 3)

To investigate the effects of Maspin on proliferation and sensitivity to cisplatin of bladder cancer T24 and 5637 cells, we used MTT and colony formation assay. The results showed both cells viability were reduced with the increasing of cisplatin concentrations. Overexpression of Maspin dramatically decreased the survival rate (Fig. 3a) of both T24 and 5637 cells as compared with that of Con and NC group (*P* < 0.05). The IC50 results showed that Maspin overexpression increased the sensitivity of both T24 and 5637 cells to cisplatin, with the IC50 value decreasing from 23.32 ± 4.30 mg/l to 15.30 ± 2.10 mg/l and from 4.91 ± 0.42 mg/l to 2.14 ± 0.21 mg/l, respectively (Fig. 3b). Also, clone-formation assay showed the cells proliferation were inhibited in Maspin group of T24 and 5637 (*P* < 0.05) (Fig. 3c, d). It suggested that Maspin could inhibit the proliferation and enhance sensitivity to cisplatin of T24 and 5637 cells.

### Maspin increase cell apoptosis induced by cisplatin in T24 and 5637 cells

**Fig. 4:**
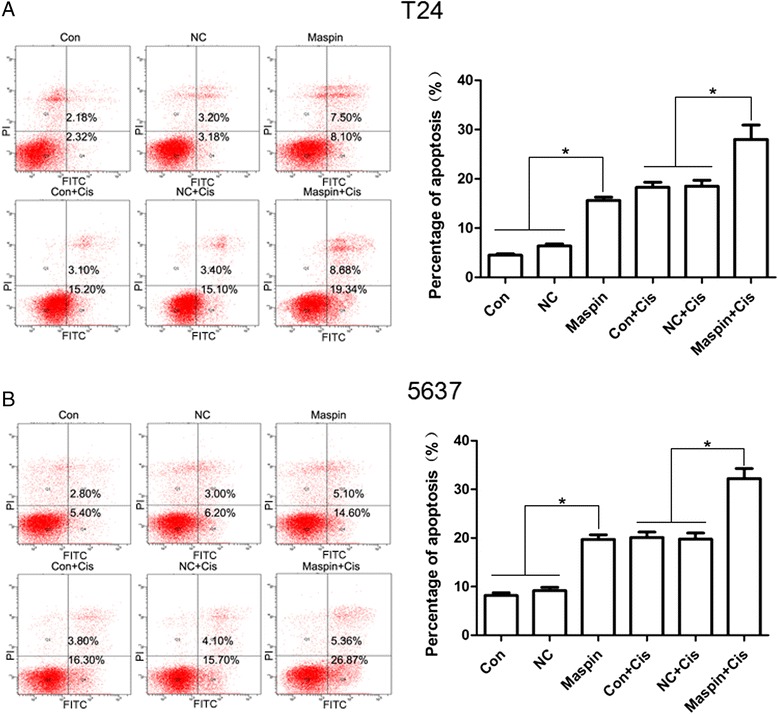
Effects of Maspin on cisplatin-induced cell apoptosis in T24 and 5637 cells. Apoptotic cells were detected by Annexin V-FITC and PI double staining and analyzed by flow cytometry. **a** and **b** were showed maspin overexpression enhanced cell apoptosis induced by cisplatin in T24 and 5637 cells, respectively. *P < 0.05. Three independent experiments were performed (*n* = 3)

To determine whether the Maspin-induced growth inhibition was mediated by apoptosis, we further used the flow cytometry to identify the cell apoptosis rate. We evaluate the influence of Maspin on T24 and 5637 cells apoptosis induced by 25 mg/L and 6 mg/L cisplatin. As showed in Fig. 4, the apoptosis rates were elevated in all group (Con, NC and Maspin) when adding the cisplatin. Overexpression of Maspin increase T24 and 5637 cells apoptosis at about 12.48 and 12.53 % (from 15.60–28.08 % and from 19.70–32.23 %), respectively. (*P* < 0.05). This was consistent with the change of drug sensitivity and demonstrated that Maspin induced chemosensitivity alteration was probably mediated by the cell apoptosis pathway.

### The probable mechanism of upregulation of Maspin enhances cisplatin-induced chemosensitivity in T24 and 5637 cells

**Fig. 5:**
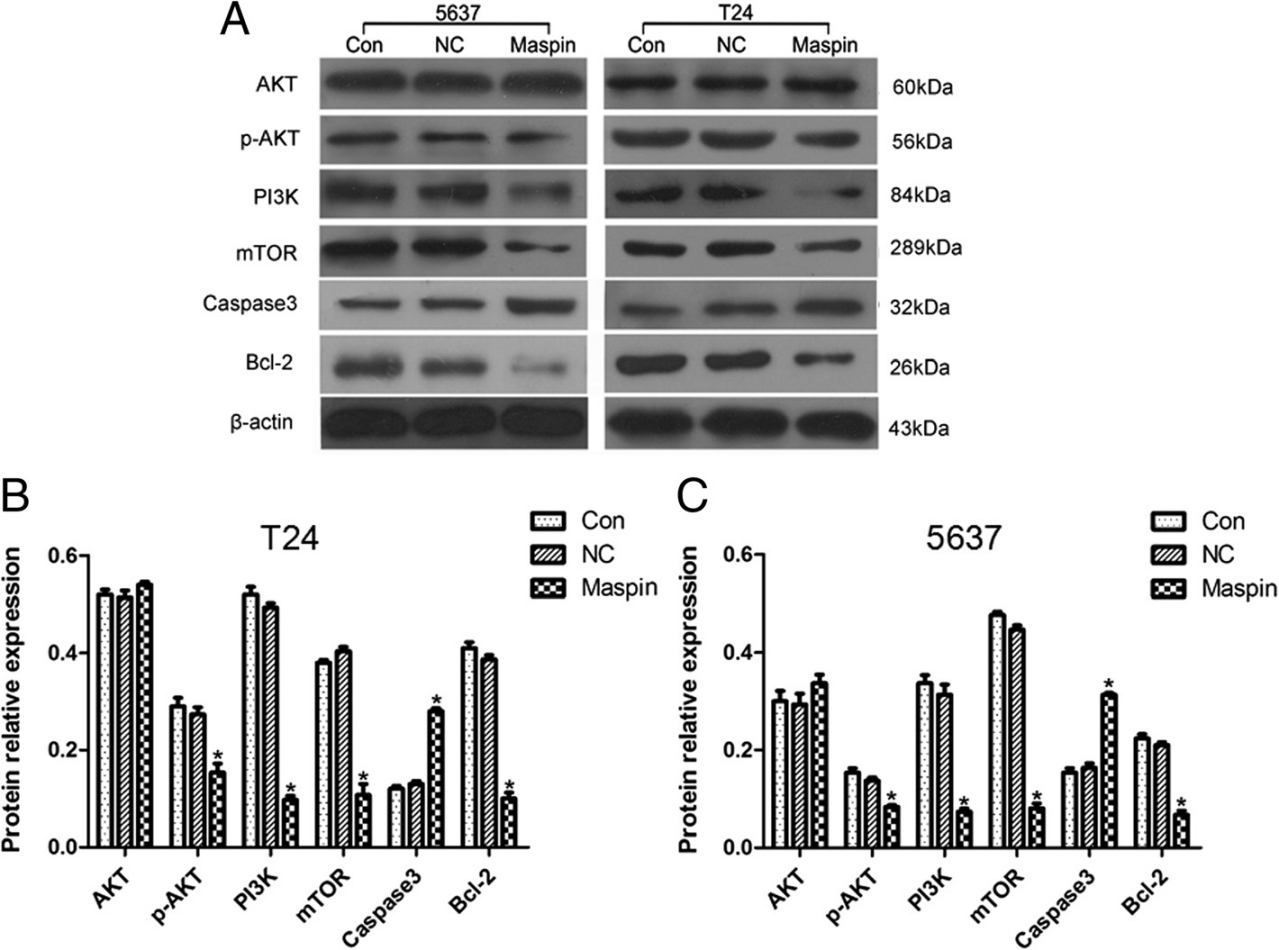
Western blotting analysis of proteins related to apoptosis in T24 and 5637 cells. **a** was showed AKT, p-AKT, PI3K, mTOR, Caspase3 and Bcl-2 expression in T24 and 5637 cells measured by immunoblotting. And β-actin was used as an internal control. **b** and **c** were showed the relative protein levels normalized to β-actin in T24 and 5637 cells, including Con, NC and Maspin group, respectively. AKT expression did’t have significant changes between each three group (*P* > 0.05). p-AKT, PI3K, mTOR, and Bcl-2 expression were significantly decreased, whereas Caspase3 was greatly increased in the Maspin group. *P < 0.05. Three independent experiments were performed (*n* = 3)

To further elucidate the probable mechanism of Maspin enhancing cisplatin-induced chemosensitivity in T24 and 5637 cells, western blotting analysis was used to examine the effects on AKT, phospho(p)-AKT, PI3K, mTOR, Caspase3 and Bcl-2 protein expressions. All groups were conducted after cells culture in IC50 cisplatin enviroment. Our results showed that PI3K, which could activate AKT, was downregulated after Maspin overexpression (*P* < 0.05). Though AKT expression did’t have significant changes between each three group (*P* > 0.05), p-AKT was decreased after Maspin overexpression (*P* < 0.05). And mTOR, a substrate of the PI3K/AKT signaling pathway, was downregulated in Maspin group (*P* < 0.05). We also found apoptosis related molecules Caspase3 was increased and Bcl-2 decreased in Maspin group (*P* < 0.05) (Fig. 5).

### Maspin expression in the MIBC patient samples

**Fig. 6:**
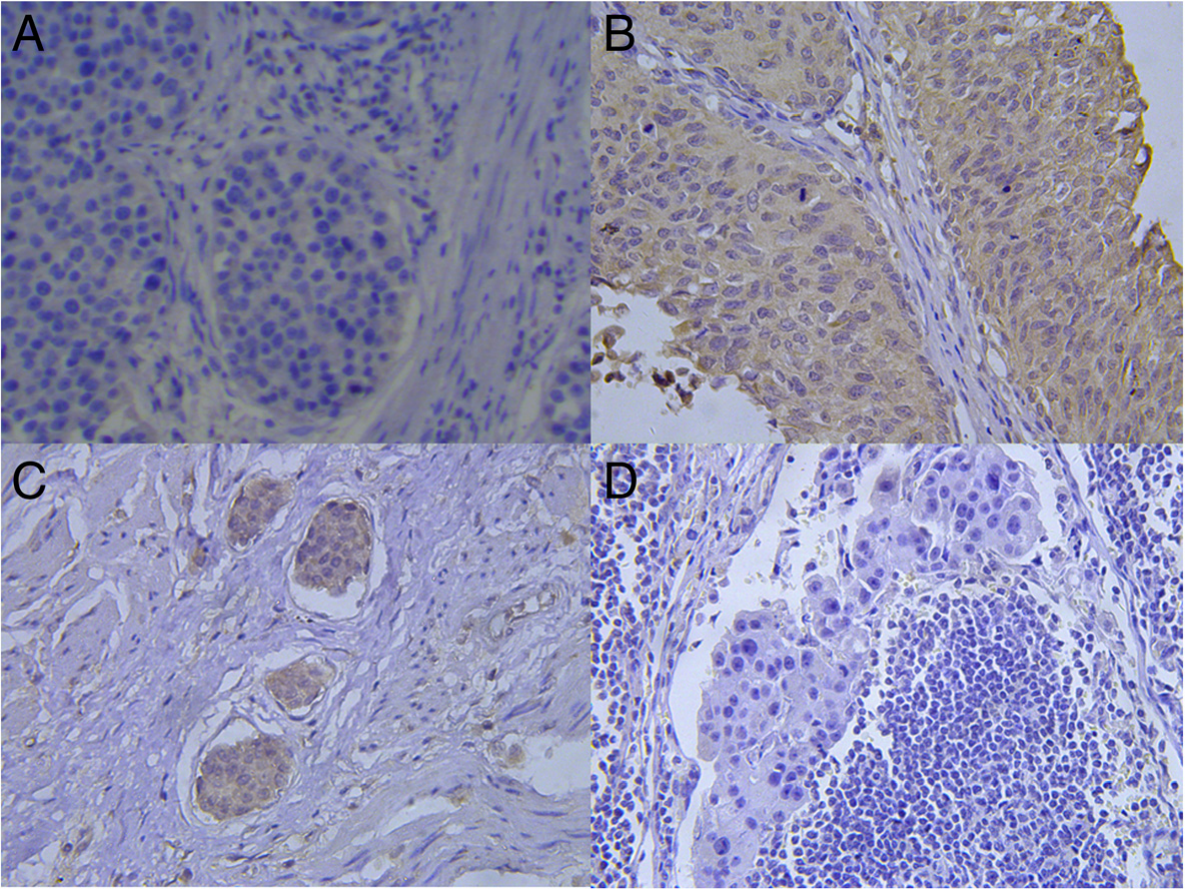
Immunohistochemistry of bladder cancer tissue with Maspin antibody. **a** showed negative(−) stain of Maspin in MIBC sample. **b** showed positive(+) stain of Maspin in MIBC sample. **c** showed bladder cancer cells invaded into micro-vessels. **d** showed the lymph node metastasis of bladder cancer cells. (**a**, **b**, **c** and **d**, magnification, ×400)

To assess Maspin expression in the samples of MIBC patient receiving cisplatin based chemotherapy, Maspin protein was detected in the nucleus and cytoplasm of the MIBC specimens. There were 23 (37.1 %) cases of Maspin (+) and 39 (62.9 %) cases of Maspin (−) in all MIBC cases (Fig. 6a, b). We also saw the micro-vessels invasion (Fig. 6c) in 41 (66.1 %) cases and lymph node metastasis (Fig. 6d) in 34 (54.8 %) cases.

### Maspin expression in correlation with the prognosis of MIBC patients receiving cisplatin based neoadjavant chemotherapy

**Fig. 7:**
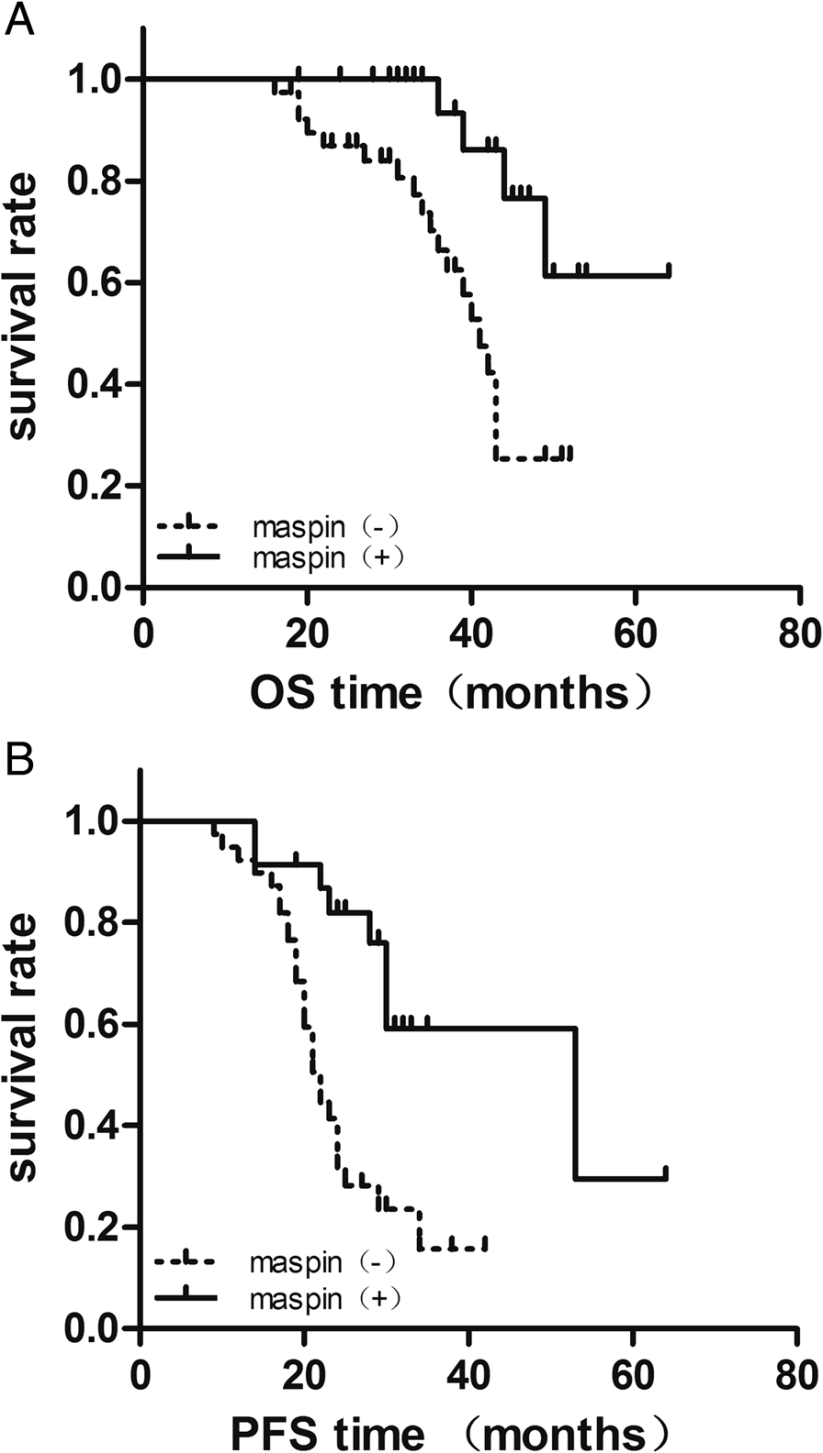
The relationship between Maspin staining and survival rate in MIBC patients receiving NACT plus RC-PLND. **a** and **b** showed Kaplan-Meier survival curves of the OS and PFS in the 62 patients, respectively. The results indicated Maspin expression was related to OS and PFS (*P* = 0.0036 and 0.0005, respectively)

To determine if Maspin was associated with the prognosis of MIBC patient receiving cisplatin based neoadjuvant chemotherapy, patients’ survival was analyzed with log-rank test (Table 2) and Kaplan-Meier survival curves (Fig. 7). In all 62 MIBC patients, the overall survival (OS) rate among the patients with Maspin (−) expression level was potentially significantly lower than that among the patients with Maspin (+) expression (*P* = 0.0036). The progression-free survival (PFS) rate among patients with maspin (−) expression level was also potentially significantly lower than that among the patients with Maspin (+) expression (*P =* 0.0005). These results showed Maspin (+) expression was correlated with good prognosis in MIBC patients who underwent cisplatin based NACT.

**Table 2:** Log-rank test of Maspin expression with MIBC survival time

Log rank (Mantel-Cox)	*χ* ^2^	95 % CI	*P* value
Maspin and OS	8.49	1.521–8.515	0.0036
Maspin and PFS	12.17	1.725–6.980	0.0005

*OS* overall survival, *PFS* progression-free survival, *CI* confidence interval. *P* < 0.05 was considered statistically significant

## Discussion

Maspin is a 42-kDa protein associated with various tumor related processes such as the inhibition of cell migration, cell invasion, angiogenesis, as well as improvement in cell adhesion and the induction of programmed cell death, thus classifying it as a tumor suppressor [15–17]. Emerging evidence shows that Maspin expression was preserved in superficial bladder cancers and its expression associated with good prognosis. Kramer [18] investigated specimens from 162 non-muscle invasive bladder cancer patients treated by transurethral resection. They showed positive Maspin expression rate was 75.9 % and had a longer PFS compared with negative expression (46 vs 18 months, respectively). And Acikalin, Blandamura also concluded similar results on Maspin expression and superficial bladder cancer [19, 20]. In our study, there were 23 (37.1 %) cases of Maspin (+) in 62 MIBC cases. The OS and PFS rate among patients with Maspin (−) were significantly lower than these among patients with Maspin (+) expression (*P* = 0.0036 and 0.0005, respectively). This expression rate was lower than the reported results in superficial bladder cancer while it correlated with the Beecken’s study [10], who showed Maspin was significantly diminished in MIBC and patients with high Maspin expression had relatively good prognosis.

Aa a tumor suppressor, Maspin has been found to increase cell apoptosis induced by chemo-regents in several types of cancers. Jiang et al. found Maspin transfected MDA-MB-435 breast carcinoma cells displayed increased sensitivity to staurosporine induced apoptosis [21]. Ben et al. testified Maspin could mediate E2F1-induced apoptosis of U2OS and SAOS2 osteosarcoma cells to doxorubicin and cisplatin [22]. Additional supporting evidence using prostate cancer cells had also reported that Maspin induced apoptosis sensitivity to chemical reagents [23], as well as increased the rate of apoptosis related to inhibition of proteosomal pathways [24].

In addition to surgical treatment, chemotherapy is an important strategy for the therapy of MIBC. Cisplatin based chemotherapy is widely used in bladder cancer treatment [25]. However, a significant proportion of patients develop disease progression after an initial response to chemotherapy. The drug resistance affect the therapeutic efficacy of bladder cancer treatment [26]. Recently, several molecules and reagents had been found to enhance chemosensitivity in bladder cancer, such as miR-101, TFAP2a, LRIG1, 5-Azacytidine, LY294002, et al. [27–32]. But the crosstalk between Maspin and cisplatin based chemosensitiviy is rare till now. In the present study, our results showed that Maspin overexpression increased the cell apoptosis and enhanced chemosensitivity of both T24 and 5637 cells to cisplatin, with the IC50 value decreasing from 23.32 ± 4.30 mg/l to 15.30 ± 2.10 mg/l and from 4.91 ± 0.42 mg/l to 2.14 ± 0.21 mg/l, respectively. Further more, in MIBC patients treating with NACT plus RC-PLND, Maspin expression was correlated with the prognosis, showing the OS and PFS rates were much longer in Maspin(+) group.

Bcl-2, a 26-kDa integral, is a powerful endogenous anti-apoptosis protein that in response to a variety of cell death signals [33]. Research showed it negatively regulated the activation of Caspase-3, the key executioner of apoptosis, which functions as an effector of mammalian cell death pathways. Downexpression of Bcl-2 promote Caspase activities and apoptosis [34]. The present study results show upregulation of the Maspin expression in T24 and 5637 cells, resulting in a decrease in the Bcl-2 level, with a concurrent increase in the Caspase-3 activation. Recent evidence indicates that AKT is frequently activated in many types of human cancer, and activated PI3K/AKT/mTOR signaling pathway is a significant contributor to tumor progression and poor prognosis, including bladder cancer [35]. Moreover, AKT overexpression is often associated with resistance to cisplatin chemotherapy, such as in ovarian and lung cancer, et al. [36, 37]. Sun et al. testified MK2206, a highly potent and selective allosteric AKT inhibitor, could promote the cisplatin-induced cytotoxicity and apoptosis in urothelial cancer cell lines through inhibiting the Akt pathway [38]. Wu and his colleagues found LY294002, a PI3K inhibitor, could suppress proliferation and sensitize doxorubicin chemotherapy in bladder cancer EJ cells. Our results showed that compared with control group, though AKT expression did’t have significant changes, p-AKT, PI3K and mTOR expression were significantly decreased in Maspin group. So, the above results suggest upregulating Maspin expression could enhance cisplatin chemosensitivity and was induced by cell apoptosis through PI3K/AKT/mTOR signaling pathway in T24 and 5637 cells.

As concluded by Berardi, there are three strategies to re-activate or increase Maspin expression [8]. The first one is designing transcription factors, to hit the system that inhibits the expression of maspin, such as artificial transcription factors (ATFs) and chromatin remodeling drugs [39]. In our previous study, we found a DNA methyltransferase inhibitor, 5-aza-2′-deoxycytidine (5-Aza-CdR), could reactivate Maspin expression and inhibit the proliferation, migration, and invasion of T24 cells, which may serve as a potential strategy for the treatment of bladder cancer [40]. The second one is identifying natural substances that can determine the activation and the expression of Maspin, such as curcumin, a hydrophobic polyphenol derived from turmeric [41]. And Last but not least, gene therapy capable of up-regulating the Maspin in cancer cell, such as Maspin DNA-liposome therapy and adeno-associated virus (AAV, serotype 2) vector encoding Maspin [42, 43].

## Conclusions

In conclusion, Maspin could enhance cisplatin chemosensitivity induced by cell apoptosis in MIBC T24 and 5637 cell lines. The probable mechanism was associated with PI3K/AKT/mTOR signaling pathway. Its expression correlated with prognosis of MIBC patients who received cisplatin-based NACT. This study provides new insight into Maspin’s role in MIBC and shows its promise as a potential molecular target for bladder cancer.

## Abbreviations

MIBC: muscle invasive bladder cancer
NACT: neoadjuvant chemotherapy
OS: overall survival
PFS: progression-free survival
RC-PLND: radical cystectomy and pelvic lymph node dissection

## References

[1] Torre LA, Bray F, Siegel RL, Ferlay J, Lortet-Tieulent J, Jemal A (2015). Global cancer statistics, 2012. CA Cancer J Clin.

[2] Herr HW (2001). Transurethral resection of muscle-invasive bladder cancer: 10-year outcome. J Clin Oncol.

[3] Botteman MF, Pashos CL, Redaelli A, Laskin B, Hauser R (2003). The health economics of bladder cancer: a comprehensive review of the published literature. Pharmacoeconomics.

[4] Cookson MS, Herr HW, Zhang ZF, Soloway S, Sogani PC, Fair WR (1997). The treated natural history of high-risk superficial bladder cancer: 15-year outcome. J Urol.

[5] Gallagher DJ, Milowsky MI, Bajorin DF (2008). Advanced bladder cancer: status of first-line chemotherapy and the search for active agents in the second-line setting. Cancer.

[6] Witjes JA, Compérat E, Cowan NC, De SM, Gakis G, Lebret T (2014). EAU guidelines on muscle-invasive and metastatic bladder cancer: summary of the 2013 guidelines. Eur Urol.

[7] Tanji N, Ozawa A, Miura N, Yanagihara Y, Azuma K, Sasaki T (2010). Long-term results of combined chemotherapy with gemcitabine and cisplatin for metastatic urothelial carcinomas. Int J Clin Oncol.

[8] Berardi R, Morgese F, Onofri A, Mazzanti P, Pistelli M, Ballatore Z (2013). Role of maspin in cancer. Clin Transl Med.

[9] Bodenstine TM, Seftor RE, Khalkhali-Ellis Z, Seftor EA, Pemberton PA, Hendrix MJ (2012). Maspin: molecular mechanisms and therapeutic implications. Cancer Metastasis Rev.

[10] Beecken WD, Engl T, Engels K, Blumenberg C, Oppermann E, Camphausen K (2006). Clinical relevance of maspin expression in bladder cancer. World J Urol.

[11] Pawel S, Verena M, Malgorzata GZ, Andrzej W, Irina K, Marek S (2006). Maspin expression is characteristic for cisplatin-sensitive ovarian cancer cells and for ovarian cancer cases of longer survival rates. Int J Gynecol Pathol.

[12] Triulzi T, Ratti M, Tortoreto M, Cristina G, Piera A, Viola R (2014). Maspin influences response to doxorubicin by changing the tumor microenvironment organization. Int J Cancer.

[13] Lei KF, Liu BY, Jin XL, Guo Y, Ye M, Zhu ZG (2012). Prognostic value of nuclear maspin expression for adjuvant 5-fluorouracil-based chemotherapy in advanced gastric cancer. Exp Ther Med.

[14] Sugimoto S, Maass N, Takimoto Y, Katsuhiko S, Sadatsugu M, Ming Z (2004). Expression and regulation of tumor suppressor gene maspin in human bladder cancer. Cancer Lett.

[15] Zou Z, Anisowicz A, Hendrix MJ, Thor A, Neveu M, Sheng S (1994). Maspin, a serpin with tumor-suppressing activity in human mammary epithelial cells. Science.

[16] Zhang M, Volpert O, Shi YH, Bouck N (2000). Maspin is an angiogenesis inhibitor. Nat Med.

[17] Lockett J, Yin S, Li X, Meng Y, Sheng S (2006). Tumor suppressive maspin and epithelial homeostasis. J Cell Biochem.

[18] Kramer MW, Waalkes S, Hennenlotter J, Serth J, Stenzl A, Kuczyk MA (2010). Maspin protein expression correlates with tumor progression in non-muscle invasive bladder cancer. Oncol Lett.

[19] Acikalin D, Oner U, Can C, Acikalin MF, Colak E (2012). Predictive value of maspin and Ki-67 expression in transurethral resection specimens in patients with T1 bladder cancer. Tumori.

[20] Blandamura S, D’Alessandro E, Giacomelli L, Guzzardo V, Battanello W, Repele M (2008). Expression of maspin in papillary Ta/T1 bladder neoplasms. Anticancer Res.

[21] Jiang N, Meng Y, Zhang S, Mensah OE, Sheng S (2002). Maspin sensitizes breast carcinoma cells to induced apoptosis. Oncogene.

[22] Ben Shachar B, Feldstein O, Hacohen D, Ginsberg D. The tumor suppressor maspin mediates E2F1-induced sensitivity of cancer cells to chemotherapy. Mol Cancer Res. 2010;8:363–372. 10.1158/1541-7786.MCR-09-0137 20197383

[23] Tahmatzopoulos A, Sheng S, Kyprianou N (2005). Maspin sensitizes prostate cancer cells to doxazosin-induced apoptosis. Oncogene.

[24] Li X, Chen D, Yin S, Meng Y, Yang H, Landis-Piwowar KR (2007). Maspin augments proteasome inhibitor-induced apoptosis in prostate cancer cells. J Cell Physiol.

[25] Costantini C, Millard F (2011). Update on chemotherapy in the treatment of urothelial carcinoma. Scientific World Journal.

[26] Galluzzi L, Vitale I, Michels J, Brenner C, Szabadkai G, Harel-Bellan A (2014). Systems biology of cisplatin resistance: past, present and future. Cell Death Dis.

[27] Ye L, Bin L, Shiyu T, Lin Q, Xiheng H, Yunbo C (2015). miR-101 suppresses vascular endothelial growth factor C that inhibits migration and invasion and enhances cisplatin chemosensitivity of bladder cancer cells. PloS One.

[28] Nordentoft I, Dyrskjøt L, Bødker JS, Wild PJ, Hartmann A, Bertz S (2011). Increased expression of transcription factor TFAP2 correlates with chemosensitivity in advanced bladder cancer. BMC Cancer.

[29] Yan Z, Jiang J, Li F, Yang W, Xie G, Zhou C (2015). Adenovirus-mediated LRIG1 expression enhances the chemosensitivity of bladder cancer cells to cisplatin. Oncol Rep.

[30] Kavitha R, Edna G, Rakesh S (2011). 5-azacytidine reverses drug resistance in bladder cancer cells. Anticancer Res.

[31] Zhou H, Tang K, Xiao H, Zeng J, Guan W, Guo X (2015). A panel of eight-miRNA signature as a potential biomarker for predicting survival in bladder cancer. J Exp Clin Cancer Res.

[32] Wu D, Tao J, Xu B, Qing W, Li P, Lu Q (2011). Phosphatidylinositol 3-kinase inhibitor LY294002 suppresses proliferation and sensitizes doxorubicin chemotherapy in bladder cancer cells. Urol Int.

[33] Hockenbery DM, Zutter M, Hickey W, Nahm M, Korsmeyer SJ (1991). BCL2 protein is topographically restricted in tissues characterized by apoptotic cell death. Proc Natl Acad Sci U S A.

[34] Gross A, McDonnell JM, Korsmeyer SJ (1999). BCL-2 family members and the mitochondria in apoptosis. Genes Dev.

[35] Sun CH, Chang YH, Pan CC (2011). Activation of the PI3K/Akt/mTOR pathway correlates with tumour progression and reduced survival in patients with urothelial carcinoma of the urinary bladder. Histopathology.

[36] Yang X, Fraser M, Moll UM, Ajoy B, Tsang BK (2006). Akt-mediated cisplatin resistance in ovarian cancer: modulation of p53 action on caspase-dependent mitochondrial death pathway. Cancer Res.

[37] Liu LZ, Zhou XD, Qian G, Shi X, Fang J, Jiang BH (2007). AKT1 amplification regulates cisplatin resistance in human lung cancer cells through the mammalian target of rapamycin/p70S6K1 pathway. Cancer Res.

[38] Sun D, Sawada A, Nakashima M, Kobayashi T, Ogawa O, Matsui Y (2015). MK2206 potentiates cisplatin-induced cytotoxicity and apoptosis through an interaction of inactivated Akt signaling pathway. Urol Oncol.

[39] Beltran A, Parikh S, Liu Y, Cuevas BD, Johnson GL, Futscher BW (2007). Re-activation of a dormant tumor suppressor gene maspin by designed transcription factors. Oncogene.

[40] Zhang H, Qi F, Cao Y, Zu X, Chen M, Li Z (2013). 5-Aza-2′-deoxycytidine enhances maspin expression and inhibits proliferation, migration, and invasion of the bladder cancer T24 cell line. Cancer Biother Radiopharm.

[41] Anand P, Sundaram C, Jhurani S, Kunnumakkara AB, Aggarwal BB (2008). Curcumin and cancer: an “old-age” disease with an “age-old” solution. Cancer Lett.

[42] Schaefer JS, Zhang M (2006). Targeting maspin in endothelial cells to induce cell apoptosis. Expert Opin Ther Targets.

[43] Watanabe M, Nasu Y, Kashiwakura Y, Nasu Y, Kashiwakura Y, Kusumi N (2005). Adeno-associated virus 2-mediated intratumoral prostate cancer gene therapy: long-term maspin expression efficiently suppresses tumor growth. Hum Gene Ther.

